# Determinants for the success of regional ICT ventures: a close examination of South Korea

**DOI:** 10.1186/s40064-016-2712-5

**Published:** 2016-07-11

**Authors:** Eunil Park, Ki Joon Kim, Sang Jib Kwon, Jay Y. Ohm, Angel P. del Pobil, Kyeongsik Yoo

**Affiliations:** Korea Institute of Civil Engineering and Building Technology (KICT), Goyang, Republic of Korea; Department of Media and Communication, City University of Hong Kong, Kowloon, Hong Kong; Department of Business Administration, Dongguk University, Gyeongju, Republic of Korea; Korea Advanced Institute of Science and Technology (KAIST), N5 2111, KAIST College Building 2, 291 Daehak-ro, Yuseong-gu, Daejeon, 34141 Republic of Korea; Department of Interaction Science, Sungkyunkwan University, Seoul, Republic of Korea; University Jaume-I, Castellón, Spain; Daejeon Technopark, Daejeon, Republic of Korea

**Keywords:** Innovation effort, Government support, Economic performance, Job opportunity, ICT venture, Daedeok Innopolis

## Abstract

**Background:**

This study identifies the key motivational factors in enhancing economic performance and increasing new job opportunities for information and communication technology ventures (ICTVs) in South Korea and examines their potential causal relationships through structural equation modeling analysis on data collected from over 200 ICTVs located in Daedeok Innopolis.

**Results:**

The results indicate that the economic performance of ICTVs is determined mainly by government support, innovation effort, and private equity and support. Government support and innovation effort are also positively associated with new job opportunities.

**Conclusions:**

The theoretical, industrial implications of the key findings, and recommendations for the Korean government are discussed.

## Background

Over the past few decades, South Korea has emerged as a leader in information and communication technology (ICT), while related businesses and ventures have grown dramatically in both quality and quantity. Extensive research on the key motivational factors for the rapid economic growth has ascertained that high-quality human resources and systematic government support have played significant roles in promoting economic growth and sustainability in South Korea (Sengupta and Espana 1994; Pahlavani and Harvie 2008; Shin and Hassink 2011). The financial and political support of the government is believed to be the leading driver of nationwide research and development (R&D) activities (Kim 1999; Yun and Lee 2013). Additionally, private equity and support as well as innovations such as user-centered organizational strategies (Becker and Dietz 2004) and internal research and development (Löfsten and Lindelöf 2005), have played equally critical roles in enhancing the economic capacity of ICT ventures (ICTVs), especially by increasing job opportunities (Bogliacino and Pianta 2010; Hall et al. 2008; Herzog 2011; Herzog and Leker 2010).

Given the importance of innovation effort, private equity and support, and government support, this study examines the effects of these motivational factors and their contribution to the success of ICTVs in South Korea. Focusing on the ICTVs located in Daedeok Innopolis, this study proposes a research model that explicates how the motivational factors help improve the economic performance of, and increase job opportunities at, ICTVs.

## Literature review and hypotheses

### History of Daejeon Daedeok Innopolis (DDI)

The South Korean government established an education and research zone in Daejeon in the early 1970s to advance its national R&D capacity. Construction of the infrastructure and research centers began in the mid-1970s. Active collaboration among academia, industry, and research institutes began in the early 1990s upon completion of the Daedeok Research Complex in 1992; the successful hosting of the 1993 Daejeon Expo encouraged many private research centers to move to DDI. To support this collaboration, the government approved the building of a technology-oriented commercialization district providing organizations relocating in DDI with easy access to a large pool of qualified scientists and researchers as well as over 25 % of the government’s entire R&D expenditure pool. In the early 2000s, legislation (e.g., the Proclamation of the Daedeok Valley, the Law of Technology Transfer Promotion, the Special Act on Developing DDI, the Special Act on Support of the Daedeok Special Research and Development Zone) was passed to support the growth of DDI (Park et al. 2011).

DDI is divided into five zones. Zone I (27.2 km^2^) and Zone V (4.9 km^2^) consist of traditional science parks, including a number of research-oriented institutes. Zones II (4.3 km^3^) and Zone III (3.1 km^2^) comprise specialized industrial complexes designed to attract high-tech and traditional companies, respectively. Zone IV (30.2 km^2^) is preserved as an undeveloped green belt zone for future use. These zones host five research-oriented universities (e.g., the Korea Advanced Institute of Science and Technology), 29 national research institutes, and more than 1000 ventures (including approximately 400 IT-based companies). Unlike the large South Korean conglomerates known as *chaebols*, most of the institutes and companies in DDI are small and medium-sized.

By 2009, more than 45,000 DDI researchers had achieved approximately 67,000 patents and 900 technology transfers. Kim and An (2012) argue that the positive impact of the DDI ICTVs on the national economy has been produced largely through government policies and programs. They also recommend that the government improve its programs for companies in other industries, such as biotechnology and nanotechnology. Given the successes of ICTVs and their expansion in DDI, investigating the critical factors in that success is worthwhile both theoretically and practically.

After the successful launch of DDI, the South Korean government decided to create similar innovation clusters. Gwangju Innopolis, Daegu Innopolis, and Busan Innopolis were established in 2011 and became local industry and economy hubs. Gwangju Innopolis develops next-generation optical convergences, environmental-friendly automobiles, smart-grids, cultural content, and bio-materials; it also aims to promote South Korea’s optics industry as the global cutting-edge in nanotechnology. Daegu Innopolis specializes in medical equipment as well as smart IT, green energy, and mechatronics convergences; it fosters the convergence of various technologies as the backbone of South Korea’s national industries. Finally, Busan Innopolis specializes in shipbuilding, offshore plant materials, offshore plat engineering and services, and green marine machinery (Jung and Mah 2014).

### Innovation effort

Innovation effort, such as extending existing knowledge and developing new technologies, has become an essential business component in a rapidly changing society (Shan et al. 1994; Cainelli et al. 2004, 2006; Wong et al. 2005). Since Schumpeter (1961) introduced the concept of “innovation,” most companies, institutes, and organizations have experimented with innovation as the growth engine for success. There are two types of innovation effort: open and closed. Chesbrough (2003, 2006) argued that open innovation effort is one of the most effects tools for firm success.

Much research has demonstrated the positive effects of both open and closed innovation effort on firms’ R&D (Caloghirou et al. 2004; Souitaris 2002; Amara and Landry 2005; Kim and Park 2010; Kang and Kang 2009). Open innovation effort, including user-centered and organizational innovation, leads to higher R&D levels (Becker and Dietz 2004; Shan et al. 1994), while closed innovation also positively affects R&D (Löfsten and Lindelöf 2005; Boscherini et al. 2012; Herzog and Leker 2010). In South Korea, Lee et al. (2010) introduced various networking models to support the view that open innovation in small and medium-sized enterprises significantly improves performance. In line with their findings, this study predicts that ICTV innovation effort is positively associated with the ventures’ economic performance and ability to create job opportunities:

#### **H1**

Innovation effort is positively associated with ICTVs’ economic performance.

#### **H2**

Innovation effort is positively associated with job opportunities.

### Private equity and support

Private equity is known to have positive effects on firms’ economic performance. Wright et al. (2009) suggest that factors related to private equity such as return to investors, profitability, and productivity positively affect firms’ economic and social conditions. Similarly, several studies (e.g., Wright et al. 2000, 2001; Cotter and Peck 2001; Guo et al. 2011; Cornelli and Karakas 2008) have demonstrated that private equity improves firm performance, especially by allowing stockholders to monitor and engage in the firm’s activities.

In addition, the positive relationship between private equity and support and employment has been frequently documented (Wood and Wright 2010). For example, Bacon et al. (2013) developed a framework for four different types of private equity, showing that private equity buyouts were positively associated with creating new job opportunities in firms. In accordance with these findings, this study predicts that private equity and support have positive effects on ICTVs’ economic performance and job opportunity creation:

#### **H3**

Private equity and support services are positively associated with ICTVs’ economic performance.

#### **H4**

Private equity and support services are positively associated with job opportunities.

### Government support

Government support is generally considered among the most important antecedents for firm success (McWilliams and Siegel 2001). Government financial and political support improves the financial stability and general condition of high-tech firms (Kang and Park 2012). Studies have investigated the role of government support, including public training and financial programs, in improving firm performance (Lerner 1996). Howe and Mcfetridge (1976) explored the effects of government support on the R&D activities of Canadian companies to determine whether it improved their performance and efficiency. Several studies (e.g., Alchian and Demsetz 1972; Oakey 1983; Levy and Terleckyj 1983; Kim 2005) have revealed notable relationships between government support and the overall productivity of national economies. Favre et al. (2002) demonstrated that the French government’s financial and political support promoted firms’ R&D activities as well as cooperation with other organizations (Favre et al. 2002). Dollar and Sokoloff (1990) found that the success and productivity of South Korean manufacturing companies were largely determined by government policies and support. Moreover, the World Bank (1993) identified government support as one of the most essential factors in the growth of companies in East Asia, South Korea, Japan, and Taiwan.

In addition, several studies have revealed positive relationships between national and local government support for companies and new employment opportunities (Klenow 1996; Lerner 1996). For example, Erickson and Friedman (1990) and Alvarez et al. (2009) showed that national government support promoted the creation of new jobs in several US states. Based on these consistent findings, this study proposes the following hypotheses on government support:

#### **H5**

Government support is positively associated with ICTVs’ economic performance.

#### **H6**

Government support is positively associated with job opportunities.

### Research model

**Fig. 1:**
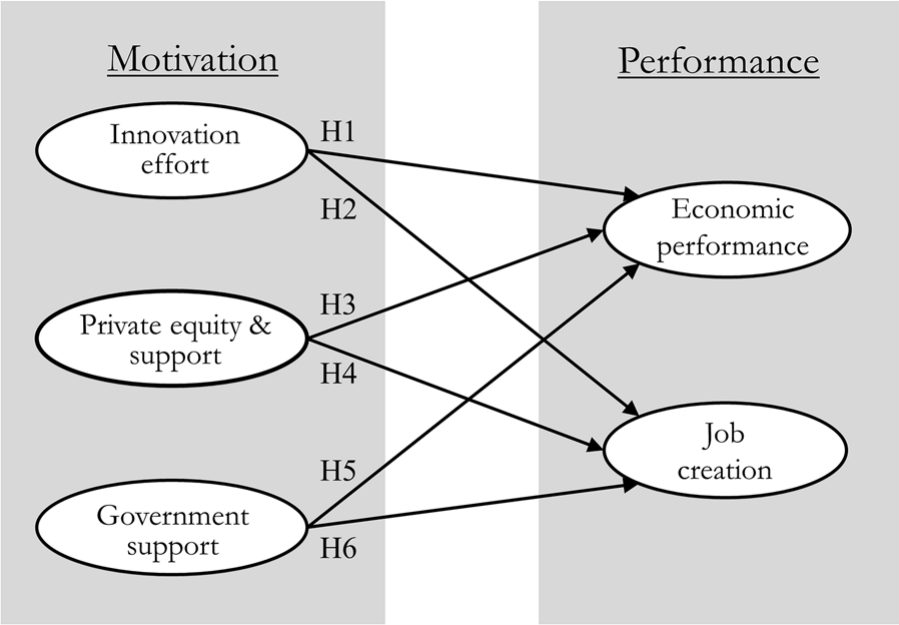
The research model

Based on the posited hypotheses and causal relationships, the research model depicted in Fig. 1 below is proposed.

## Study design

### Data collection

This study used the 2012 Daejeon Regional Economic Reviving Survey conducted by Daejeon Technopark (a local government institute). The survey database contained information about companies located in the Daejeon metropolitan area since 2011, including their R&D activities, economic performance, number of employees, and current status. This study identified potential motivational antecedents through 10-min in-depth interviews with the managers of 20 ICTVs before administering the main survey. Using the interview results, this study determined the critical factors in ICTVs’ economic performance (see Table 1). The main survey was sent to 300 venture companies drawn from the database. After excluding the companies that did not complete the survey, 213 companies remained as the final sample.

**Table 1:** Results of in-depth interviews for identifying potential antecedents

	Factors	N (%)
1	Government support	30 (37.0 %)
2	Innovation effort	22 (27.2 %)
3	Private equity and support services	12 (14.8 %)
4	Merger and acquisition	7 (8.6 %)
5	Product and service diversification	4 (4.9 %)
6	Etc.	6 (7.4 %)
Total		81 responses from 20 managers^a^

^a^Multiple responses were allowed

### Measured variables

The construct of innovation effort was measured with three items adopted from Evangelista et al. (2001). Private equity and support were measured with three items adopted from Dakhli and De Clercq (2004) and Luk et al. (2008). Government support was assessed with three items adopted from Cai et al. (2010). Economic performance was assessed with three items used in Henri and Journeault (2010) and Skiba et al. (2009). Job creation was measured with three items adopted from Lester (2005) and Kwon et al. (2015). A complete list of the questionnaire items used in this study appears in Table 2.

**Table 2:** Questionnaire items used in this study

Construct	Item
Innovation effort	
IE1	My company has invested adequate innovation efforts in internal and external R&D
IE2	My company has invested adequate innovation efforts in manufacturing, services, and production
IE3	My company has invested adequate innovation efforts in design and marketing
Private equity and support	
PE1	The private equity and support services have positive effects on the quality of products and services offered by the company
PE2	The private equity and support services have positive effects on the financial conditions of the company
PE3	The private equity and support services are considered important components of the company
Government support	
GS1	Government support has positive effects on the quality of the products and services offered by the company
GS2	R&D programs supported by the government have positive effects on efficiency and the current status of the company
GS3	Supports (e.g., technological assistance) provided by the government have positive effects on efficiency and the current status of the company
Economic performance	
EP1	The sales rate of the company has improved
EP2	The return on assets (ROA) and return on sales (ROS) of the company have improved
EP3	The operating profits and cash flow of the company have improved
Job creation	
JC1	The number of new job has increased
JC2	The quality of new entry job positions has improved
JC3	There will be a notable increase in the creation of new employment opportunities in the company

### Data analysis

A confirmatory factor analysis (CFA) and structural equation modeling (SEM) using the LISREL 8.70 software were conducted to examine the validity of the measurement model and proposed research model, respectively. Research has found that SEM requires the minimum sample size to be larger than 200 for analytical validity (Fornell and Larcker 1981; Hair et al. 2006). The sample size of this study (*N* = 213) meets this criterion.

## Results

### Measurement model

As summarized in Table 3, the overall fit indices of the measurement model were satisfactory, except the ratio of the Chi square to the degrees of freedom (χ^2^/*df*). Values for composite reliability and Cronbach’s alpha were calculated to test the validity of each construct. Prior studies recommend that all factor loadings and composite reliability values exceed 0.50 and 0.70, respectively (Anderson and Gerbing 1988; Hair et al. 2006). All correlations between constructs should be lower than the values of the square root of the average variance extracted (Fornell and Larcker 1981). This study’s measurement model satisfied all these standards (see Tables 4, 5).

**Table 3:** The fit indices of the measurement model

Fit indices	Values	Recommended level	Source
χ^2^/*df*	4.66 (*p* < 0.01)	<3.0	Bagozzi and Yi (1988)
NFI	0.955	>0.900	Bentler and Bonnett (1980)
IFI	0.911	>0.900	Browne and Cudeck (1993)
CFI	0.924	>0.900	Fornell and Larcker (1981)
GFI	0.912	>0.900	Hair et al. (2006)
AGFI	0.901	>0.900	Hoe (2008)
SRMR	0.040	<0.050	Holbert and Stephenson (2002)
RMSEA	0.041	<0.050	Jöreskog and Sörbom (1996)

*NFI* normed fit index, *IFI* incremental fit index, *CFI* comparative fit index, *GFI* goodness-of-fit index, *AGFI* adjusted goodness-of-fit index, *SRMR* standardized root mean square residual, *RMSEA* root mean square error of approximation

**Table 4:** Internal validity and convergent reliability of the constructs

Construct	Item	Internal validity		Convergent reliability		
		Cronbach’s alpha	Item-total correlation	Factor loadings	Composite reliability	Average variance extracted
Innovation effort	IE1	0.869	0.778	0.914	0.921	0.794
	IE2		0.847	0.871		
	IE3		0.822	0.889		
Private equity and support services	PE1	0.871	0.812	0.896	0.921	0.795
	PE2		0.806	0.899		
	PE3		0.836	0.880		
Government support	GS1	0.770	0.663	0.845	0.868	0.686
	GS2		0.732	0.802		
	GS3		0.676	0.837		
Economic performance	EP1	0.817	0.844	0.786	0.891	0.733
	EP2		0.709	0.881		
	EP3		0.675	0.897		
Job creation	JC1	0.838	0.751	0.884	0.903	0.756
	JC2		0.758	0.879		
	JC3		0.813	0.845

**Table 5:** Results of discriminant validity; diagonal elements are the square root-values of the average variance extracted

Construct	1	2	3	4	5
1. Innovation effort	0.891				
2. Private equity and support services	0.108	0.892			
3. Government support	0.229	0.185	0.828		
4. Economic performance	0.388	0.079	0.402	0.856	
5. Job creation	0.321	0.255	0.391	0.224	0.869

### Hypotheses testing

The hypotheses were tested by validating the structural model. The SEM results indicated that the overall fit indices of the proposed research model were satisfactory, except χ^2^/*df* (see Table 6).

**Table 6:** The fit indices of the research model

Fit indices	Values	Recommended level	Source
χ^2^/*df*	4.97 (*p* < 0.01)	<3.0	Bagozzi and Yi (1988)
NFI	0.936	>0.900	Bentler and Bonnett (1980)
IFI	0.901	>0.900	Browne and Cudeck (1993)
CFI	0.919	>0.900	Fornell and Larcker (1981)
GFI	0.921	>0.900	Hair et al. (2006)
AGFI	0.925	>0.900	Hoe (2008)
SRMR	0.048	<0.050	Holbert and Stephenson (2002)
RMSEA	0.047	<0.050	Jöreskog and Sörbom (1996)

*NFI* normed fit index, *IFI* incremental fit index, *CFI* comparative fit index, *GFI* goodness-of-fit index, *AGFI* adjusted goodness-of-fit index, *SRMR* standardized root mean square residual, *RMSEA* root mean square error of approximation

**Fig. 2:**
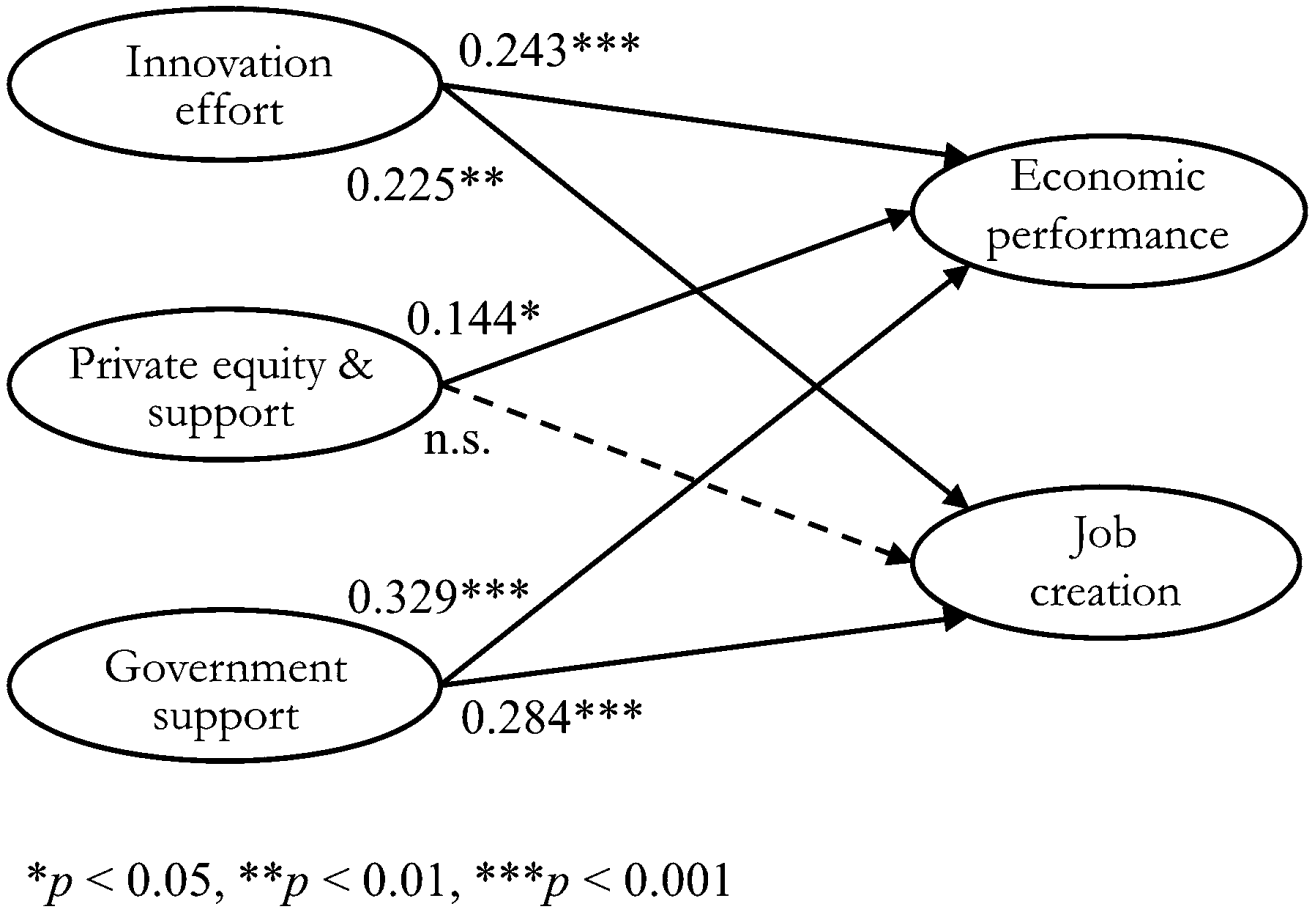
Summary of the research model

As Fig. 2 and Table 7 show, all hypotheses were supported except H4. The economic performance of ICTVs was determined by government support (H5, *β* = 0.329, *p* < 0.001), innovation effort (H1, *β* = 0.243, *p* < 0.001), and private equity and support (H3, *β* = 0.144, *p* < 0.05). New job opportunities were influenced by two factors—government support (H6, *β* = 0.284, *p* < 0.001) and innovation effort (H2, *β* = 0.225, *p* < 0.01). However, private equity and support did not have a significant effect on job creation (H4, *p* > 0.05); 24.2 % of the variance in job creation was explained by innovation effort and government support, while government support, innovation effort, and private equity and support explained 28.8 % of the variance in ICTVs’ economic performance.

**Table 7:** Results of the hypothesis tests

Hypothesis	Path coefficient	Standard error	Critical ratio	Supported
H1. Innovation effort → Economic performance	0.243***	0.062	3.408	Yes
H2. Innovation effort → Job creation	0.225**	0.089	3.214	Yes
H3. Private equity and support services → Economic performance	0.144*	0.057	1.917	Yes
H4. Private equity and support services → Job creation	0.096	0.082	1.309	No
H5. Government support → Economic performance	0.329***	0.074	3.991	Yes
H6. Government support → Job creation	0.284***	0.052	4.495	Yes

*** *p* < 0.001; ** *p* < 0.01; * *p* < 0.05

## Discussion

This study proposed and validated an integrated research model for economic performance and job creation to examine the role of innovation effort, private equity and support, and government support in enhancing ICTVs’ economic performance and capacity to offer jobs. Our findings suggest that innovation effort and government support are the most efficient motivational factors in the successful growth of ICTVs, thus rejecting the null hypotheses.

These results of our SEM analysis provide several noteworthy implications for researchers and practitioners. This study offers a systematic and comprehensive understanding of a structural concept concerning three motivations for and two outputs of the economic performance and job creation of ICTVs in DDI. The SEM results confirm that innovation effort is not the only important factor in increasing firms’ economic performance but that both government support and private equity and support are also significant determinants of ICTVs’ economic performance. Innovation effort and government support are also revealed as key determinants of job creation.

Second, our findings provide meaningful insights into ways of facilitating the plans and operations of South Korean ICTVs. The South Korean economy is heavily reliant on manufacturing, and much of the government’s support is devoted to promoting the infrastructure and hardware aspects of innovation clusters.

## Conclusions

The current study explores the core motivations in improving economic performance for ICTVs in South Korea. Based on the structural results from the data of more than 200 ICTVs, several key points can be presented.

Based on the findings, the current study provides several insights for South Korean ICT industry. The Korean government should aim to provide carefully planned political, financial, and physical assistance to bolster the software aspects of innovation, such as human resources, finance, and R&D collaboration (Park et al. 2014). Specifically, Table 8 shows the recommendations for the Korean government which should place greater emphasis.

**Table 8:** Recommendations for the Korean government

Order	Content
1	Integrating the nation’s support systems
2	Integrating government departments and agencies to increase communication efficiency
3	Investing in R&D and human resources rather than providing direct financial support
4	Strategic planning for ICTV-specific support policies
5	Providing prompt administrative assistance
6	Providing appropriate tax support
7	Designing effective curricula for IT personnel training
8	Expanding collaboration between industry and academia
9	Employing experienced retirees
10	Establishing collaborative research facilities
11	Developing region-specific facilities and services

The relatively weak effects of private equity and support might have been produced by circumstances specific to South Korea’s ICT industry. Private equity and support are uncommon in South Korea, and less social capital and support are available than government support; thus, most ICTVs may not require the benefit of social capital and support. This suggests that the Korean government should expand its support by providing the equivalent of private equity and support.

This study has several limitations. First, generalizing our findings to other regions or countries is difficult because our sample is restricted to ICTVs in one area of South Korea. Second, several unexamined factors might have affected the proposed causal relationships in the research model. Studies have found that cultural and organizational factors (Casson 1993; Hansen and Warnerfelt 1989) and environmental disclosure (Al-Tuwaijri et al. 2004) have significant effects on firms’ economic performance. Additionally, while the global ICT industry typically specializes in both hardware and software, ICTVs in South Korea focuses primarily on the hardware sector of the ICT industry, thereby restricting the generalizability of our findings. By addressing these limitations, future studies may develop a more comprehensive model for predicting the economic performance of ICTVs at the international level.
